# Selective Reduction of Multiple Pregnancies Into Singleton Pregnancy: Treatment Analysis for Fetal Position

**DOI:** 10.1155/jp/6113048

**Published:** 2026-06-24

**Authors:** Haiyan Liu, Run Chen, Xuan Huang, Yi Zhou, Yanmin Luo, Zhuyu Li

**Affiliations:** ^1^ Department of Obstetrics and Gynecology, The First Affiliated Hospital of Sun Yat-sen University, Guangzhou, Guangdong, China, sysu.edu.cn; ^2^ Guangdong Provincial Clinical Research Center for Obstetrical and Gynecological Diseases, Guangzhou, Guangdong, China

**Keywords:** dichorionic triamniotic pregnancy, fetal reduction, miscarriage, monochorionic diamniotic trichorionic triamniotic pregnancy, preterm birth, triplet pregnancies

## Abstract

**Background:**

The rise in assisted reproductive technology has increased the prevalence of high‐risk triplet pregnancies. Dichorionic triamniotic (DCTA) triplets—where a monochorionic‐diamniotic (MCDA) twin pair shares a placenta—carry significant risks, including twin‐to‐twin transfusion syndrome. Although reducing a multifetal pregnancy to a singleton via MFPR leads to better outcomes, how the location of the reduced fetus within the uterus affects the success of the pregnancy is not addressed in current clinical guidance.

**Objectives:**

This study compares obstetric outcomes based on the location of the reduced MCDA twin pair (superior vs. inferior position) in DCTA triplet pregnancies.

**Methods:**

A retrospective analysis was conducted on 200 patients who underwent MFPR to a singleton between 2013 and 2024. Patients were divided into Group A (MCDA pair located farther from the cervix/superior) and Group B (MCDA pair located closer to the cervix/inferior). Primary outcomes included miscarriage rates (before 24 weeks) and gestational age at delivery.

**Results:**

Group A demonstrated a significantly lower miscarriage rate (4.1% vs. 17.9%, *p* = 0.001) and a higher mean gestational age at delivery (38.38 vs. 37.36 weeks, *p* = 0.015) compared to Group B. Additionally, the incidence of low birth weight was significantly lower in Group A (10.3% vs. 26.6%, *p* = 0.004). No significant differences were found in perinatal death or Apgar scores between the groups.

**Conclusions:**

In DCTA pregnancies, reducing the MCDA twin located farther from the cervix is associated with lower miscarriage rates and improved gestational outcomes.

Why Was This Study Conducted?

To the best of our knowledge, there have been no studies on fetal reduction conducted in a cohort of all multiple pregnancies, comparing the positions of fetuses in diverse orientations.

Key Findings

Fetal reduction performed far from the cervix is a safe procedure with a low risk of miscarriage or stillbirth compared to procedures performed close to the cervix. When fetal reduction is performed close to the cervix, the risk of adverse pregnancy outcomes and preterm delivery is lower than when the procedure is done before 14 weeks.

What Does This Study Add to What Is Already Known?

Our results support fetal reduction performed far from the cervix.

## 1. Introduction

Multiple pregnancy, defined as the presence of two or more fetuses in the uterus, is relatively rare in the general population. In recent years, rising maternal age, widespread use of ovulation induction drugs, and advances in assisted reproductive technology (ART) have contributed to a marked increase in its incidence [1, 2], as well as a growing economic burden [3].

Triplet pregnancies carry a particularly high risk, with maternal and fetal complication rates occurring more than six times as often as in singleton pregnancies. Among triplet gestations, dichorionic triamniotic (DCTA) pregnancies are the second most common variety. These pregnancies present unique clinical challenges because two of the fetuses—the monochorionic‐diamniotic (MCDA) twin pair—share a single placenta. This shared placental architecture makes them highly susceptible to complex vascular complications, such as twin‐to‐twin transfusion syndrome (TTTS) and selective intrauterine growth restriction (sIUGR)

[4–6]. To manage these risks, multifetal pregnancy reduction (MFPR) is often employed to prolong the gestational period and improve overall outcomes. Studies have shown that reducing a DCTA pregnancy to a singleton significantly increases the likelihood of term delivery, with mean gestational ages reaching 38 weeks, while simultaneously reducing the costs associated with neonatal intensive care [7–18]. Current clinical guidelines, including China′s “Technical Standards for Multifetal Pregnancy Reduction,” emphasize that when managing DCTA pregnancies, priority should be given to reducing the MCDA pair to a singleton to eliminate shared placental risks [19]. Despite this consensus, there is a notable lack of evidence regarding the optimal technical approach to fetal selection based on intrauterine position [2, 16]. While general recommendations suggest avoiding the reduction of a fetus located close to the internal cervical os to minimize the risk of infection, however, comparative data on how these different locations affect miscarriage rates and long‐term pregnancy success are still absent [20].

Consequently, there is a critical need for clinical evidence to guide physicians in determining the safest location for reduction. This retrospective cohort study aims to compare pregnancy outcomes based on the intrauterine location of the reduced MCDA twin pair in DCTA triplet pregnancies. By evaluating the disparities between reductions performed in the superior (far from the cervix) and inferior (close to the cervix) positions, we seek to provide valuable evidence for clinical risk assessment and the optimization of MFPR procedures.

## 2. Materials and Methods

### 2.1. Study Population and Design

This retrospective cohort study was conducted from January 2013 to January 2024. The study population comprised patients carrying DCTA triplets who voluntarily underwent MFPR to a singleton at the First Affiliated Hospital of Sun Yat‐sen University. Among the women who were included in this study, three live fetuses and chorionicity (DCTA) were confirmed by ultrasound examination. Cases were included in this study if they met all of the following criteria: DCTA triplet pregnancy, maternal age ≥ 18 years, gestational age at the time of reduction was subsequent to the completion of nuchal translucency (NT) screening, fetal reduction performed with the specific selection to reduce the MCDA twin pair, the remaining singleton fetus exhibited normal amniotic fluid volume and no evidence of structural malformations or developmental abnormalities, absence of maternal contraindications for fetal reduction, and no clinical manifestations of miscarriage or chorioamnionitis prior to the procedure. The exclusion criteria are as follows: following MFPR, termination of pregnancy may be required due to retained fetal chromosomal or genetic abnormalities; severe fetal structural malformations or other reasons; and incomplete medical records.

All patients received comprehensive counseling detailing the risks of a triplet pregnancy and the risks and benefits of MFPR. Following this, each participant provided written informed consent. The indications for MFPR included (1) uncomplicated triplet pregnancies and (2) pregnancies with abnormalities in one or more fetuses, such as severe structural anomalies on ultrasound or thickened NT.

To account for the possibility of spontaneous fetal reduction and early ultrasound limitations, the MFPR procedure—intracardiac injection of potassium chloride (KCl) into one fetus of the MCDA pair—was performed after the NT ultrasound examination, thereby reducing the DCTA pregnancy to a singleton. Under continuous ultrasound guidance, the fetal heart was successfully accessed with a single puncture.

As for the perioperative management, prophylactic oral antibiotics and tocolytics were administered perioperatively. Cephalosporins were the first‐line antibiotics due to their broad spectrum and favorable safety profile [21, 22]. Specifically, cefuroxime (250 mg) was administered orally 1 h preoperatively and continued twice daily for 24 h postoperatively, with monitoring of infection markers. We typically conducted a follow‐up blood count the next day. If maternal peripheral blood white blood cell count was (≥15 × 10^9^/L), but without evidence of clinical chorioamnionitis, the use time of antibiotics would be prolonged or even intravenous administration would be extended. If maternal peripheral blood white blood cell count became normal, we routinely discontinued antibiotics. Considering cross‐allergic reaction, for patients with a penicillin allergy, azithromycin was used [23, 24].

Clinical chorioamnionitis [25–27] was defined as maternal temperature ≥ 38°C plus one or more of the following: maternal tachycardia (≥ 100 bpm) or fetal tachycardia (baseline ≥ 160 bpm), uterine tenderness, malodorous vaginal discharge, or maternal leukocytosis (≥15 × 10^9^/L), after excluding other infectious etiologies.

The procedure was executed by or under the guidance of fetal medicine consultants, each possessing extensive experience in invasive prenatal diagnostic techniques. All patients underwent an ultrasound examination either 1 day or 1 week following the procedure, in order to confirm the viability of the remaining fetus. If one of the MCDA pregnancy cardiac activities was identified, the reduction surgery will be repeated on the same day, provided that the condition has been thoroughly explained to the pregnant woman and her family, and their informed consent has been obtained.

According to the 2025 ISUOG Clinical Practice Guidelines [28], following independent assessment and consensus reached by two or more experienced sonographers, the “cervix‐proximal group” is defined as a fetal presentation located inferiorly on the sagittal plane, and the “cervix‐distal group” is defined as a fetal presentation located superiorly on the sagittal plane. Hence, based on the fetal position determined by real‐time ultrasound scanning during selective reduction surgery, these patients were divided into two groups: Group A, in which the MCDA pair was located farther from the cervix (superior), and Group B, in which the MCDA pair was located closer to the cervix (inferior).

### 2.2. Outcome Measures

The primary outcomes were (i) the rate of miscarriage, which is defined as pregnancy loss before 24 weeks of gestation, and (ii) the survival rates of preterm birth after 28 weeks of gestation. Secondary outcomes included preterm birth (< 37 completed weeks), very preterm birth (< 32 completed weeks), low birth weight (LBW; 1500 to < 2500 g), very low birth weight (VLBW; < 1500 g), and other relevant metrics. Gestational age at delivery was determined using a hierarchical algorithm based on the date of the last menstrual period, earliest ultrasound findings, NT measurement, or details from assisted reproduction.

### 2.3. Statistical Analysis

All statistical analyses were performed using SPSS Version 26.0 (IBM SPSS Statistics Inc.). Categorical variables were summarized as frequencies and percentages (%). Continuous variables were summarized as mean ± standard deviation or median (interquartile range) based on their distribution as assessed by normality tests. Between‐group comparisons were made using the two independent samples *t*‐test or the Mann–Whitney *U* test for continuous variables, and the chi‐square test or Fisher′s exact test for categorical variables. All tests were two‐sided, and a *p* value of ≤ 0.05 was considered statistically significant. Furthermore, to visualize the distribution of gestational age at delivery (unadjusted), we used the Kaplan–Meier method to plot survival curves (with delivery as the endpoint event) and applied the log‐rank test to compare differences between groups. We constructed logistic regression models to assess the association between the site of fetal reduction and the risk of miscarriage as a pregnancy outcome. In order to control the potential confounding factors, we performed multivariate regression analysis, with results presented as odds ratios (OR) and their 95% confidence intervals (CI). To assess the robustness of our findings, we performed a sensitivity analysis using inverse probability of treatment weighting (IPTW) based on propensity scores. Inverse probability weights were then calculated as 1/(propensity score) for the Group B and 1/(1−propensity score) for the Group A. A weighted logistic regression model, incorporating these weights, was used to estimate the treatment effect on the primary outcome.

## 3. Results

**Figure 1:**
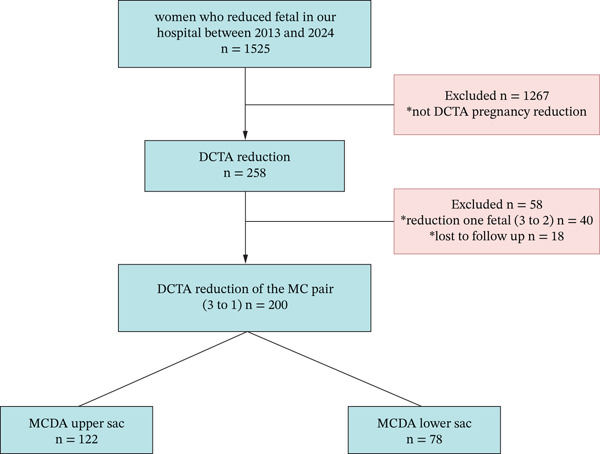
Selection of study cohort.

In total, 200 cases met the inclusion criteria between 2013 and 2024. These 200 DCTA triplet pregnancies underwent fetal reduction via intracardiac injection of KCl targeting one or both MCDA twins. Full outcome measures were available for all cases. The cohort comprised 122 cases where the MCDA twin was located far from the cervix (Group A) and 78 cases where it was located close to the cervix (Group B) (Figure 1).

Within each main group, patients were further divided by gestational age at the time of the procedure. Group A was subdivided into cases undergoing reduction before 14 weeks (Group A1) and at or after 14 weeks (Group A2). Group B was subdivided using the same criteria (Group B1 and B2, respectively).

There were no significant differences in maternal, preoperative, or operative variables between Group A and Group B (Tables 1 and 2). Specifically, there were no significant differences in mean maternal age, parity, rate of ART use, primary indication for MFPR, or gestational age at reduction.

**Table 1:** Maternal demographic and clinical characteristics.

Characteristic	A (*n* = 122)	B (*n* = 78)	*p*
Median maternal age, years (range)	31 (20–53)	32 (23–42)	0.164
Median BMI before pregnancy (range)	20.96 (16.67–30.04)	21.965 (16.02–29.73)	0.254
Nulliparous, *n* (%)	73 (59.84%)	56 (71.79%)	0.085
DCDA in early pregnancy, *n* (%)	12 (9.83%)	8 (10.26%)	0.923
Spontaneous pregnancy, *n* (%)	11 (9.01%)	7 (8.97%)	0.992
ART, *n* (%)	89 (72.95%)	55 (70.51%)	0.708
Fetal ultrasound normal request for MFPR, *n* (%)	100 (81.97%)	59 (75.64%)	0.280

*Note:* A: MCDA far from the cervix (MCDA upper sac). B: MCDA close to the cervix (MCDA lower sac).

Abbreviations: ART, assisted reproduction technique; MFPR, multifetal pregnancy reduction.

**Table 2:** Operation variables.

	A (*n* = 122)	B (*n* = 78)	*p*
Median GA at fetal reduction (range)	12 (11 + 2–22 + 1)	13 (11 + 2–22 + 2)	0.409
Fetal reduction weeks < 14 weeks (%)	95/122 (77.87%)	60/78 (76.92%)	0.870
Reduction again	6/122 (4.91%)	5/78 (6.41%)	0.652
Interval time between subsequent reductions, days (range)	3.5 (1–8)	4 (1–5)	0.464
MCDA; all had been injected with potassium chloride (%)	9/122 (7.38%)	11/78 (14.10%)	0.122

*Note:* A: MCDA far from the cervix (MCDA upper sac). B: MCDA close to the cervix (MCDA lower sac).

Abbreviations: GA, gestational age; MCDA, monochorionic diamniotic.

The obstetric outcomes and infant characteristics for the two groups were summarized in Table 3. The mean gestational age at delivery was significantly higher in Group A than in Group B (38.38 ± 1.88 weeks, 95% CI: 38.04–38.73 vs. 37.36 ± 3.01 weeks, 95% CI: 36.61–38.01; *p* = 0.015). Rates of both very preterm and preterm birth were lower in Group A. The incidence of LBW was also significantly lower in Group A (10.3%) than in Group B (26.6%; *p* = 0.004). In contrast, the rate of a 5‐min Apgar score ≤ 7 and the rate of perinatal death did not differ significantly between the groups. No neonatal deaths occurred among the remaining infants following reduction. Furthermore, there were no postoperative maternal complications such as coagulation abnormalities (disseminated intravascular coagulation [DIC]), cardiac toxicity (arrhythmia and cardiac arrest), or deaths. The miscarriage rate was significantly lower in Group A (5/122, 4.1%) than in Group B (14/78, 17.9%; *p* = 0.001). Logistic regression analysis indicates that the location of fetal reduction is an independent risk factor for miscarriage as a pregnancy outcome (OR = 5.351, 95% CI: 1.364–21.001, *p* = 0.016). To account for potential confounding factors, we performed a multivariate binary logistic regression analysis, and the selection of covariates for adjustment was based on clinical experience and published literature, after adjusting for age, body mass index, ART, gestational age at reduction, fetal abnormalities, and puncture status during reduction. This association remained statistically significant (adjusted OR = 6.533, 95% CI: 1.362–31.338, *p* = 0.019). To assess the robustness of the results, we conducted a sensitivity analysis using propensity score inverse probability weighting. The results showed that, compared with the group undergoing cervical‐distant reduction, the group undergoing cervical‐proximal reduction had a significantly higher risk of adverse pregnancy outcomes (OR = 9.85; 95% CI: 5.65–17.18; *p* < 0.001). This finding is consistent with the results of the previous unweighted multivariate regression analysis, indicating that the study results are robust to the method of adjustment for confounding factors. Although we adjusted for key confounding factors such as maternal age and gestational age at reduction, residual confounding due to unmeasured variables (such as anatomical factors and socioeconomic status) cannot be completely ruled out due to the inherent limitations of the study design. Nevertheless, the rates of miscarriage due to intrauterine infection and the incidence of intrauterine infection itself have no statistically significant difference between the two groups.

**Table 3:** Obstetric outcomes.

Perinatal outcome	A (*n* = 122)	B (*n* = 78)	*p*
Mean GA at delivery (95% CI)	38.38 ± 1.88 (38.04–38.73)	37.36 ± 3.01 (36.61–38.01)	0.015
Miscarriage rate	5/122 (4.%)	14/78 (17.9%)	0.001
Miscarriage due to intrauterine infection	1/5	3/14	0.950
Incidence of intrauterine infection	1/122	3/78	0.140
Survival	117/122 95.9% (92.3–99.5)	64/78 82.1% (73.3–90.8)	0.001
Live‐born ≥ 32 weeks	116/117 99.1% (97.5–100)	59/64 92.2% (85.4–98.9)	0.022
Live‐born ≥37 weeks	104/117 88.9% (83.1–94.7)	49/64 76.6% (65.9–87.2)	0.028
Low birth weight (< 2500 g)	12/117 10.3% (4.7–15.8)	17/64 26.6% (15.4–37.7)	0.004
Neonatal female sex	52/117	35/64	0.188
Apgar score ≤ 7 at 5 min	1/117	3/64	0.127
Neonatal complications	2/117	6/64	0.024
Perinatal death	0	0	1.000
Postoperative maternal complications or death	0	0	1.000

*Note:* A: MCDA far from the cervix (MCDA upper sac). B: MCDA close to the cervix (MCDA lower sac).

**Figure 2:**
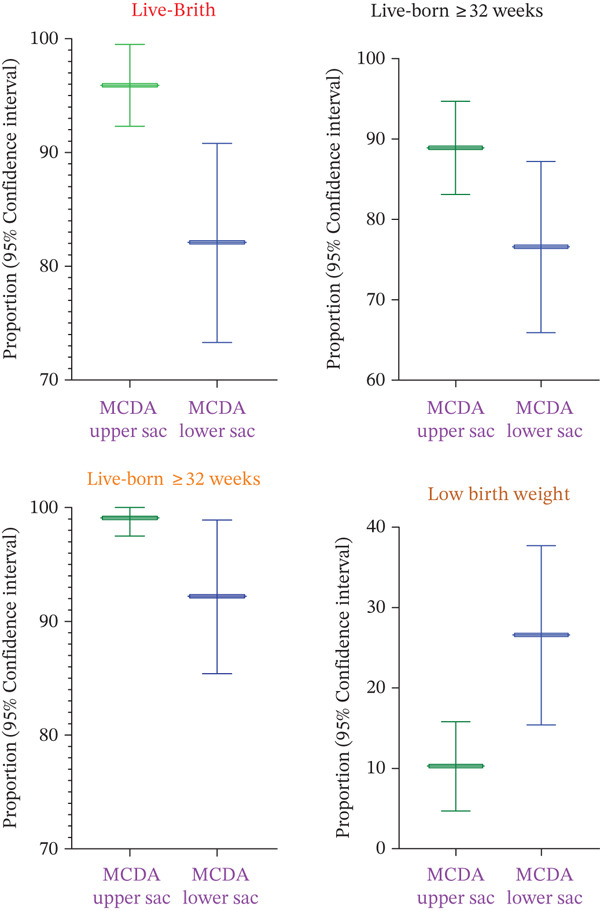
Rates of live‐born children and preterm deliveries.

**Figure 3:**
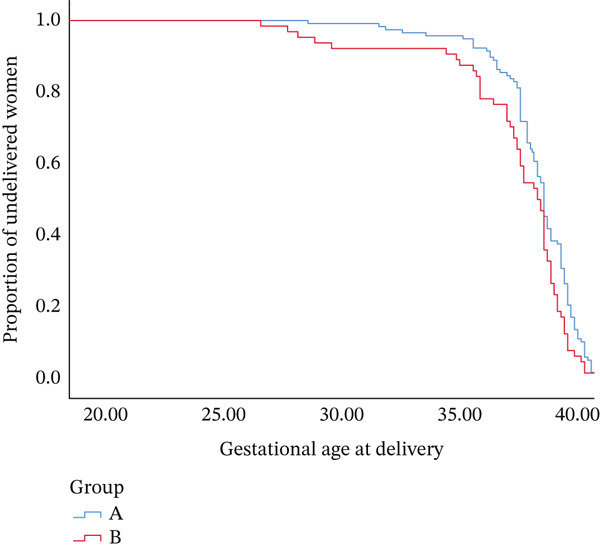
Presents time to delivery in the two different groups.

**Figure 4:**
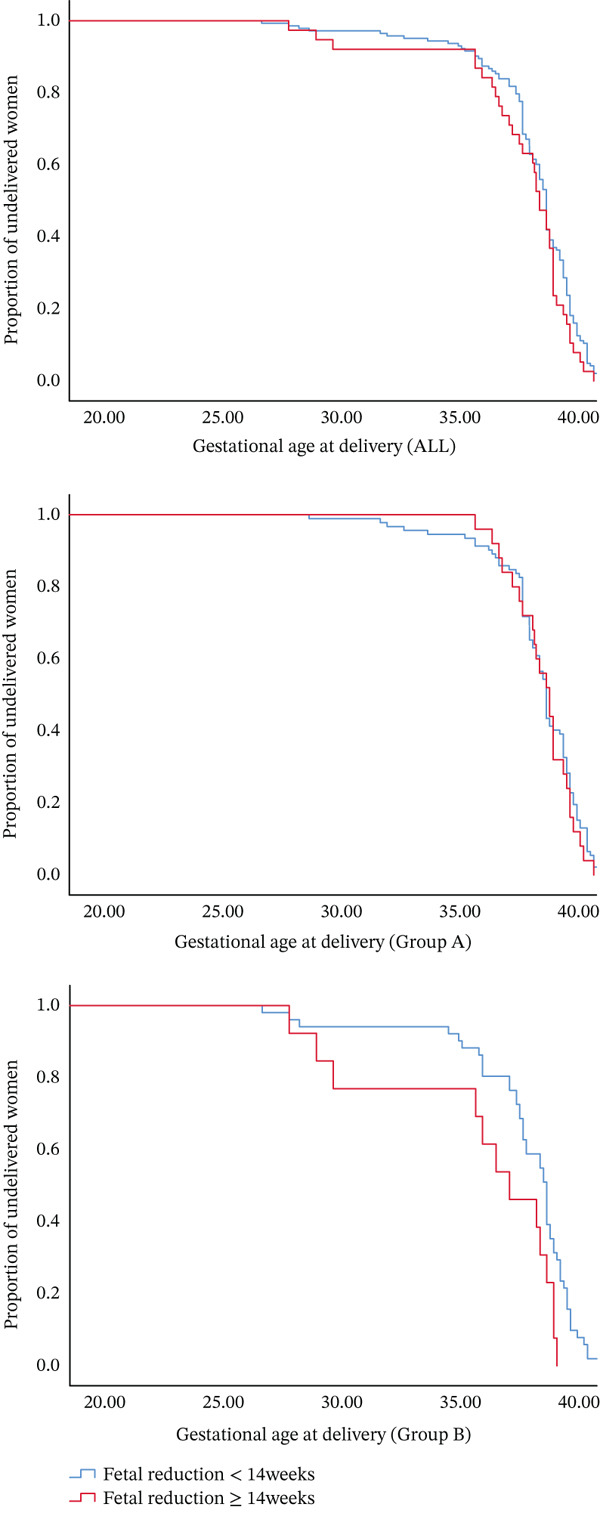
The relation between time of reduction and time to delivery.

Table 3 and Figure 2 present the rates of live birth and preterm delivery. Figure 3 also illustrates the time to delivery for each group, analyzed using Kaplan–Meier survival curves. Figure 4 demonstrates the relationship between gestational age at reduction and subsequent time to delivery. In the primary analysis, no significant difference in gestational age at birth was observed between early and intermediate reduction timings. However, upon subgroup analysis (Table 4), the gestational week of fetal reduction does indeed affect the gestational week at delivery for Group B. Miscarriage rate did not differ for time of reduction (Table 5).

**Table 4:** Comparison of outcomes between groups B1 and B2.

	B1 (*n* = 60)	B2 (*n* = 18)	*p*
Median GA at delivery	38.43	36.15	0.02
Live‐born ≥ 28 weeks	51/60	13/18	0.292
Live‐born ≥ 32 weeks	48/60	10/18	0.037
Live‐born ≥ 37 weeks	41/60	7/18	0.024

**Table 5:** The different timing of fetal reduction.

	Miscarriage rate of fetal reduction weeks < 14 weeks	Miscarriage rate of fetal reduction weeks ≥ 14 weeks	*p*
A	3 (3/95)	2 (2/27)	0.306
B	9 (9/60)	5 (5/18)	0.292
All	12/155 (7.7%)	7/45 (15.5%)	0.120

*Note:* A: MCDA far from the cervix (MCDA upper sac). B: MCDA close to the cervix (MCDA lower sac).

## 4. Discussion

In this study, the overall miscarriage rate following intracardiac KCl reduction of one or more MCDA twins in DCTA triplet pregnancies was found to be 9.5%. This finding is consistent with rates reported in prior literature on MFPR [9, 29–33].

Analysis of this cohort study indicated that during early pregnancy (prior to 10 weeks), the ultrasound examination misdiagnose rate was approximately 10% of DCTA pregnancies as dichorionic diamniotic (DCDA). This diagnostic limitation can be mitigated by the subsequent use of NT ultrasound. MFPR is usually performed between 11 and 14 weeks of gestation, as it is a period of a lower miscarriage rate (5.4%) compared to the risk of spontaneous miscarriage (12%) [34]. Hence, approximately 70% of fetal reduction procedures were performed prior to 14 weeks in this study. This practice aligns with established guidelines, whereas the observed variability likely reflects individual clinical circumstances and physician judgment within the specific context of this institution.

More favorable outcomes were observed when the superiorly located MCDA twin was selected for reduction, including a lower risk of miscarriage. A higher proportion of pregnancies reaching at least 32 weeks of gestation was achieved in this group compared to those where the inferiorly located twin was reduced. For Group A (far from cervix), a mean gestational age at delivery of 38.38 weeks and a survival rate of 95.9% were recorded, whereas for Group B (close to cervix), the corresponding figures were 37.36 weeks and 82.1%. Maternal and neonatal outcomes were favorable across all cases.

However, no significant difference was identified in the incidence of postoperative intrauterine infection based on the location of the reduced MCDA twin. This discrepancy from clinical consensus [16, 19] may be explained by the routine administration of prophylactic oral antibiotics in the present study. The background use of routine antibiotics in both groups may have masked potential intergroup differences. Due to the absence of a nonantibiotic control group and the fact that data were not collected according to a predefined, rigorous definition of infection, we cannot draw definitive conclusions regarding the efficacy of antibiotic prophylaxis. Consequently, this interpretation should be regarded as hypothesis generating, intended to provide a framework for future prospective studies. Therefore, when performing MFPR on an MCDA twin near the cervix, it is essential to employ judicious timing, refined technique, and strict aseptic precautions to mitigate the risk of uterine cavity contamination. Besides, employing pharmacological prophylaxis and enhancing postoperative care, including vigilant management of postoperative vaginal bleeding, are instrumental in effectively reducing miscarriage rates. Ultrasound‐guided early reduction and postoperative antibiotic administration remain crucial strategies in this regard.

Within this cohort, the miscarriage rate for reductions performed in early pregnancy was marginally lower (7.7%) than for those after 14 weeks (15.5%). However, there was no significant difference found in the gestational age at the time of reduction for DCTA pregnancies. Whereas some studies report no difference between early and late interventions [29, 35, 36], others describe contrasting outcomes [37–39], indicating that the current evidence is conflicting. Further studies are needed to resolve this discrepancy. It should be noted that the lack of statistical significance in this finding may be due to the limited sample size in the intermediate reduction group or owing to the fact that nearly all procedures (99%) were performed prior to 20 weeks of gestation.

Subgroup analysis revealed a key modifier: For procedures performed close to the cervix (Group B), completion of the reduction before 14 weeks was associated with a lower risk of adverse pregnancy outcomes and preterm delivery. This association may be mediated by reduced uterine overdistension at an earlier gestational age, thereby lowering the mechanical predisposition to miscarriage or preterm birth. Furthermore, an earlier procedure also allows for optimized nutrient allocation and space for the remaining fetus while reducing placental strain.

A significantly lower incidence of LBW was documented in pregnancies where the distant MCDA twin was reduced (10.3%) versus those where the proximate twin was reduced (26.6%). Neonatal complications were reported in two cases in Group A, in which both involved respiratory distress syndrome (RDS), and one along with concomitant infection. While there were six cases with neonatal complications in Group B, all six cases were RDS, along with additional complications: two with patent ductus arteriosus (PDA) and patent foramen ovale (PFO), two with gastrointestinal bleeding, one with intracranial hemorrhage (ICH), and one with neonatal infection. Nevertheless, there was no significant difference between the groups in the rate of a 5‐min Apgar score of ≤ 7 or in perinatal mortality. These comparable outcomes in critical neonatal markers may be attributable to advances in neonatal intensive care, including technological improvements in diagnostics and resuscitation, as well as the fetal lung‐maturating effects of antenatal corticosteroids such as dexamethasone administered for preterm birth risk.

## 5. Strengths and Limitations

The key strength of this study is that it is the first and largest to specifically compare pregnancy outcomes following KCl reduction of the MCDA twin at different intrauterine locations in DCTA triplet pregnancies. Several limitations should be acknowledged. First, the retrospective design may introduce selection bias and limits data validity due to potential incompleteness or inaccuracies in medical records. Second, residual confounding is possible, as important variables such as parental education and socioeconomic status were not available in our dataset. Finally, our analysis lacks long‐term neonatal follow‐up data, which restricts our ability to assess developmental outcomes beyond the perinatal period.

## 6. Conclusion

In this study of DCTA pregnancies, a higher gestational age at delivery and a lower miscarriage rate were observed when the MCDA twin located farther from the cervix was selected for reduction, compared to reduction of the closer twin. If reduction near the cervix is necessary, performing the procedure before 14 weeks appears to mitigate the risk of adverse outcomes. These findings provide valuable evidence to inform clinical risk assessment and decision‐making in MFPR.

**Nomenclature**
DCTA: dichorionic triamniotic
MCDA: monochorionic diamniotic
TCTA: trichorionic triamniotic
TTTS: twin‐to‐twin transfusion syndrome
sIUGR: selective intrauterine growth restriction
SPR: spontaneous pregnancy reduction
MFPR: multifetal pregnancy reduction
NT: nuchal translucency
RDS: respiratory distress syndrome
PDA: patent ductus arteriosus
PFO: patent foramen ovale
ICH: intracranial hemorrhage
GA: gestational age
ART: assisted reproduction technique

## Author Contributions

Haiyan Liu: conceptualization, investigation, methodology, supervision, validation, writing – original draft, writing – review and editing. Run Chen: methodology, supervision, writing – review and editing. Xuan Huang: data curation, investigation. Yi Zhou: data curation, investigation. Yanmin Luo: data curation, investigation, methodology. Zhuyu Li: conceptualization, investigation, project administration, supervision, writing – review and editing. Haiyan Liu and Run Chen contributed equally to this work.

## Funding

No funding was received for this manuscript.

## Disclosure

All authors read and approved the final manuscript.

## Ethics Statement

The First Affiliated Hospital of Sun Yat‐sen University approved the study (No. [2024]682). The study was conducted according to the Declaration of Helsinki principles. Each participant provided written informed consent to undergo the MFPR procedure.

## Consent

The authors have nothing to report.

## Conflicts of Interest

The authors declare no conflicts of interest.

## Data Availability

A part of datasets analyzed during this study are included in this published article. The rest datasets generated or analyzed during the current study are not publicly available due to patient privacy concerns, but they can be obtained from the corresponding author upon a reasonable request.
